# Epidemiological and viral studies of rabies in Bali, Indonesia

**DOI:** 10.14202/vetworld.2023.2446-2450

**Published:** 2023-12-20

**Authors:** Wayan Masa Tenaya, Nyoman Suartha, Nyoman Suarsana, Made Damriyasa, Ida Ayu Pasti Apsasi, Tri Komala Sari, Luh Putu Agustini, Yuli Miswati, Kadek Karang Agustina

**Affiliations:** 1 Department of Disease Prevention, Veterinary Public Health, Faculty of Veterinary Medicine, Udayana University, Denpasar Bali of Indonesia, Jl. PB Sudirman, Denpasar, Bali, 80234, Indonesia; 2 Department of Veterinary Clinic, Faculty of Veterinary Medicine, Udayana University, Denpasar Bali of Indonesia, Jl. PB Sudirman, Denpasar, Bali, 80234, Indonesia; 3 Laboratory of Biochemistry, Faculty of Veterinary Medicine, Udayana University, Denpasar Bali of Indonesia, Jl. PB Sudirman, Denpasar, Bali, 80234, Indonesia; 4 Laboratory of Clinical Pathology, Faculty of Veterinary Medicine, Udayana University, Denpasar Bali of Indonesia, Jl. PB Sudirman, Denpasar, Bali, 80234, Indonesia; 5 Laboratory of Parasitology, Faculty of Veterinary Medicine, Udayana University, Denpasar Bali of Indonesia, Jl. PB Sudirman, Denpasar, Bali, 80234, Indonesia; 6 Laboratory of Virology, Faculty of Veterinary Medicine, Udayana University, Denpasar Bali of Indonesia, Jl. PB Sudirman, Denpasar, Bali, 80234, Indonesia; 7 Laboratory of Virology, Veterinary Disease Investigation Center, Denpasar Bali, Jl. Raya Sesetan No. 266, Denpasar, Bali, 80223, Indonesia; 8 Laboratory of Virology, Veterinary Disease Investigation Center, Bukittinggi Jl. Bukittinggi-Payakumbuh, Tabek Panjang, Sumatra Barat, Sumatra, 26192, Indonesia; 9 Department of Disease Prevention, Veterinary Public Health, Faculty of Veterinary Medicine, Udayana University, Denpasar Bali of Indonesia, Jl. PB Sudirman, Denpasar, Bali, 80234, Indonesia.

**Keywords:** Bali, dogs, polymerase chain reaction, rabies virus

## Abstract

**Background and Aim:**

Rabies has been endemic in Bali since 2009, and cases has recently increased. Unfortunately, there is a lack of available vaccines, which hinders the eradication program. This study aimed to investigate the epidemiological and virological aspects of rabies infection in Bali.

**Materials and Methods:**

A total of 24 brain samples were collected from rabid dogs in all districts of Bali. The samples were tested using the direct fluorescent antibody (DFA) test and polymerase chain reaction (PCR) to confirm the presence of rabies virus in the samples. Samples with the highest virus content were propagated *in vivo* and then inoculated into BALB/c mice. The brains of dead mice were used to prepare an inoculate cultured in murine neuroblastoma cells. Supernatant-positive viruses representing each district were then reinoculated into eight groups of five BALB/c mice. A brain sample from each dead mouse was tested using DFA and PCR and detected under a fluorescence microscope.

**Results:**

All rabies virus-positive samples collected from rabid dogs in all districts of Bali were positive. Rabies virus was detected by DFA test and PCR and was consistently confirmed in the *in vivo* and *in vitro* studies. BALB/c mice inoculated with the highest viral dilution (105 cells/mL) of culture supernatant showed typical signs of rabies, indicating that the virus could be properly investigated.

**Conclusion:**

This study demonstrated a wide epidemiological distribution of rabies in Bali. The obtained virus can be adapted for *in vitro* and *in vivo* studies and can be used to develop a homologous vaccine.

## Introduction

Rabies is a zoonotic and potentially fatal disease in animals and humans and remains a serious health threat in many parts of the world, killing approximately 59,000 people every year [1]. In Bali, Indonesia, rabies was first reported at the end of 2008 and is now endemic [2]. In 2010, the local and central governments of Indonesia attempted to control the disease on the island and succeeded in reducing human and animal cases by 99% and 90%, respectively, between 2011 and 2013 [3, 4]. However, in 2014 and 2015, there was an increase in rabies cases in both humans and animals. In 2014, there were three deaths in humans and another 15 in 2015; in 2015, 529 cases of animal rabies were reported [5].

The elimination of rabies in Bali from 2008 to 2015 has been considered unsuccessful mainly due to the use of parenteral vaccines rather than oral vaccines promoted in other countries [6–8]. However, the use of oral vaccines in Bali and other Indonesian islands remains controversial. According to a 2011 surveillance report, although dogs were vaccinated against rabies, 12.5% of 88 brain samples from rabid dogs were positive for the rabies virus (unpublished data). Due to catastrophic conditions in 2014 and 2015, the Bali Government, supported by the Central Government, vaccinated over 400,000 dogs in all 716 villages across Bali Island in 2016 using high-quality parenteral vaccine [4]. Consequently, there was a significant decrease in animal (87%, from 529 cases to 65 cases) and human cases (100%, from 15 cases to 0 cases) cases.

In the last three years, however, since the COVID-19 pandemic, there has been a significant increase in positive cases throughout the island. During the COVID-19 pandemic, vaccination against rabies has not been carried out, and there is no vaccine available. It is likely that this situation has contributed to the reduction of immunity against rabies in dogs. In view of the limited production of rabies vaccines during the COVID-19 pandemic and budgetary considerations, it is challenging to procure internationally recommended vaccines. Therefore, isolation of the local rabies virus strain is crucial for the development of a homologous vaccine against rabies in Bali.

Advances in molecular technology have greatly contributed to the diagnosis of rabies, including the direct fluorescent antibody (DFA) test [9, 10], polymerase chain reaction (PCR) [11], and virus sequencing [12, 13]. In our study, DFA and qPCR tests were implemented to diagnose rabies in dogs in the region of the Disease Investigation Center (DIC), Denpasar. Positive DNA samples of rabies virus isolated within Indonesia were compared based on sequencing and phylogenetic analysis, which showed a high homology of 98%–99% [14]. These data suggest that the rabies virus detected in Bali has the potential to be used as a homologous vaccine candidate with a wide range of immunity, at least within Indonesia. Therefore, it is crucial to culture the rabies virus as it is the most reliable way to study rabies infection [15]. In this regard, we aimed to identify the epidemiological spread of rabies virus in Bali as an early step toward developing a homologous vaccine candidate that may be useful in some parts of Indonesia.

## Materials and Methods

### Ethical approval

This study was approved by the ethical commission of Udayana University with letter No. B/217/UN14.2.9/PT.01.04/2022.

### Study period and location

This study was conducted from March 2021 to July 2022. Samples were collected from all districts in the province of Bali, Indonesia, and laboratory work was performed at the DIC, Denpasar Bali.

### Viral detection and *in vivo* propagation

A total of 24 brain samples from rabid dogs were collected from all districts of Bali, of which three samples were collected from each district. The samples were brought to DIC Denpasar for DFA testing based on standard protocol [16] with minor modifications. In brief, each brain sample was applied to a glass object, air-dried for 5 min, and fixed with cold acetone for 15 min. After three washes with phosphate-buffered saline (PBS), the samples were incubated at 37°C for 30 min in the dark with goat anti-mouse conjugated with rabbit fluorescent isothiocyanate (Bio-Rad, California, USA). Samples were washed again with PBS and allowed to dry before buffered glycerin was dropped onto the glass surface of the object. The samples were examined under 100 fluorescence microscopy (Nikon, Japan) and then photographed.

Direct fluorescent antibody-positive samples were propagated in mice for *in vivo* viral studies, as described in the recent World Organization for Animal Health (WOAH) manual [16], with slight modifications. A 10% (w/v) fresh dog brain suspension was prepared in Dulbecco’s Modified Eagle Medium (DMEM, Thermo Fisher Scientific, Massachusetts, USA) without antibiotics and filtered through a 0.22-m MF Millipore membrane filter (Merck, USA). Eighteen 4-week-old BALB/c mice were used in this study. After anesthesia, 15 mice were intracerebrally inoculated with 10 μL of the brain suspension from each sample. The remaining two mice were infected with a challenge rabies virus standard (ATCC VR 959 control viral standard [CVS]-11), kindly provided by Dr. Enuh (BPMSOH-Bogor, Indonesia), as a positive control. A negative control was provided by inoculating five 4-week-old BALB/c mice with DMEM containing no virus using the same method described above. All the experimental mice were inspected daily. Mice that died with typical signs of rabies within 2 weeks post-viral inoculation were collected aseptically and maintained at −80°C until further use.

### *In vitro* adaptation of rabies virus

Murine neuroblastoma (N2A) cells used in this study were kindly provided by the Australian Animal Health Laboratory (AAHL, Gelong, Australia). This experiment was performed according to a previously published method [17] with minor modifications. The cells were first grown in a tissue culture flask (Iwaki, Japan) using complete DMEM (CDMEM, Thermo Fisher Scientific, Massachusetts, USA) containing 0.1 mL of heat-inactivated fetal calf serum, 2 g/mL of gentamicin, and 0.3 g/mL of amphotericin B to obtain approximately 6.0 × 10^5^ cells/mL. Cultures were incubated at 37°C in 7% CO_2_ and checked daily for contamination. Confluent cells were then harvested, and 1 mL of the cells were dropped into a 24-well tissue culture plate. We placed a sterile cover slip on the bottom of the plate and incubated the plate until the monolayer stage. The culture supernatant was aspirated, and 0.5 mL of a 10-fold dilution of the mouse brain suspension filtered through a 0.2 M filter was dropped into consecutive wells, shaken gently, and incubated at 37°C for 20 min for viral absorption. After viral absorption, the supernatant containing the virus was aspirated, and 5 mL of CDMEM was added into each well. The plate was further incubated until cytotoxic effects (CPEs) appeared. Culture supernatants of infected cells showing strong CPEs were collected and stored at −80°C for further study. Coverslips containing infected cells were fixed with acetone and detected using the standard DFA test. A rabies-infected mouse brain sample was also sent to the National Rabies Reference Laboratory at Investigation Center Bukit Tinggi, Sumatra Barat, for further confirmation using a technique similar to the DFA test.

### Confirmation of the presence of adapted virus in tissue culture

The presence of rabies virus in the culture supernatant was demonstrated *in vivo*. The methodology was similar to that described above, except that the supernatant of the rabies-infected cells was used. A tube of frozen-infected culture stored at −80°C was carefully thawed and serially diluted from 10-1 to 10^-7^ cells/mL. Ten microliters of the corresponding diluted virus was then inoculated into eight groups of five 4-week-old BALB/c mice. A negative control was provided by inoculating five BALB/c mice with 10 μL of CDMEM containing no antigens. Infected mice were checked daily without touching to observe the typical signs of rabies. A brain sample from each dead mouse was tested using DFA and detected under a fluorescence microscope (Nikon). Frozen-infected culture tubes were also sent to the National Rabies Reference Laboratory at Investigation Center Bukit Tinggi, Sumatra Barat, for polymerase chain reaction (PCR) analysis. To detect rabies virus in the cells, the coverslips with rabies-infected cells fixed with cold acetone were subjected to DFA testing.

## Results

**Figure-1:**
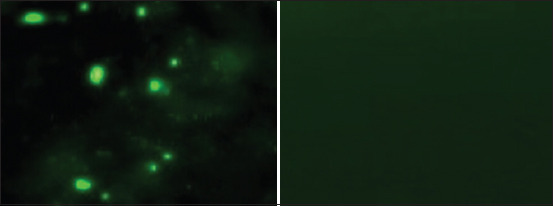
Direct fluorescent antibody test positive of the rabid dogs’ brain sample was indicated by sharp green fluorescent reactions (left), but no reaction was detected for the normal control (right), 100× magnification.

All 24 brain samples from rabid dogs originating from eight districts of Bali tested positive for the rabies virus through DFA test (Table-1); however, only ten samples revealed strong positive fluorescence signals. We selected these samples for inoculation (Figure-1).

**Table-1:** Direct fluorescent antibody test positive rabies from eight different districts in Bali with varied scores.

District origins	Total samples	DFA test results
Singaraja	3	2 (+++ ), 1 (++)
Karangasem	3	1 (+++), 2 (++)
Klungkung	3	1 (+++), 2 (+)
Bangli	3	1 (+++), 2 (++)
Gianyar	3	2 (+++), 1 (++)
Badung	3	1 (+++), 2 (+)
Tabanan	3	1 (+++),2 (++)
Jembrana	3	1 (+++), 2 (+)
Total	24	

DFA: Direct fluorescent antibody, +++: strong positive reactions, ++: mild positive reactions, +: low positive reactions

**Figure-2:**
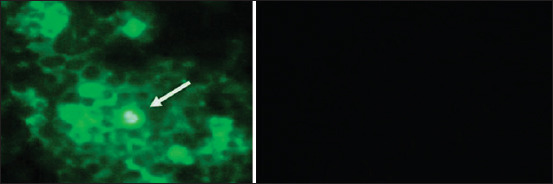
Rabies virus-infected murine neuroblastoma cells positive direct fluorescent antibody test with bright fluorescent inclusions in the cytoplasm (arrowed, left). The normal controls were negative (right), 100× magnification.

All 15 mice infected with the dog brain suspension, as well as the control-CVS-infected mice, died with typical rabies symptoms 14–16 days after infection. The negative control mice did not show any symptoms until the end of the experiment. The N2A cells infected with the brain suspensions of the dead mice showed specific CPEs (not shown) and were positive in the DFA test with a strong reaction, whereas the non-infected cells were negative (Figure-2). All naive mice inoculated with 10^5^ cells/mL of rabies culture supernatant died with classic signs of rabies similar to those observed in the positive CVS control group (data not shown).

**Figure-3:**
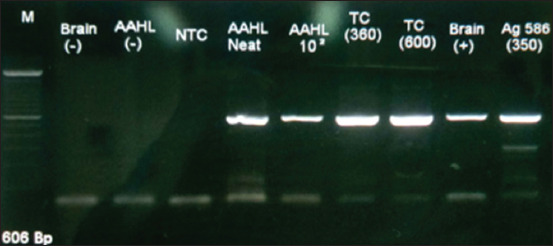
Agarose gel electrophoresis of polymerase chain reaction products amplified from the rabies-infected mice brains and tissue culture supernatants samples showed specific DNA amplifications of approximately 498 bp (lanes 2–4 from the right). The DNA amplified samples were in line with DNA positive control (Australian Animal Health Laboratory samples and Ag 568), but all negative controls did not show any amplified DNAs.

A mouse brain sample and a sample of tissue culture supernatants were tested using conventional PCR to further confirm that the samples tested positive in the DFA test contained specific rabies virus. This study was conducted at the DIC, Bukittinggi, North Sumatra, National Rabies Reference Laboratory of Indonesia. Both samples showed a very specific reaction in line with a DNA positive control provided by the AAHL (Figure-3).

## Discussion

A catastrophic increase in the incidence of rabies in Bali since 2008 has resulted in the death of hundreds of people and thousands of dogs. The fight against rabies in Bali is based on the mass vaccination of dogs and the control of the dog population; however, the results have not been satisfactory. The failure of the rabies control program in Bali is believed to be due to the lack of world-standard vaccines and insufficient control of the dog population [7]. When WOAH-recommended anti-rabies vaccines were used during the first eradication campaign (2011–2013), there was a significant reduction in both human and animal cases [3, 4]. However, due to the limited supply of these vaccines, cases of rabies in humans and animals has recently begun to increase [3, 18]. It is crucial to provide locally isolated rabies viruses to develop appropriate and effective vaccines to support the global target “zero human deaths from dog-mediated rabies” [19]. However, to prepare an alternative endogenous and homologous virus for a vaccine candidate, no significant culture work has been performed to evaluate the natural behavioral and epidemiological distribution of the virus. Therefore, epidemiological and viral studies of rabies in Bali were conducted in this study. Based on *in vitro* and *in vivo* studies, the results of the present study suggest that the rabies viruses obtained from all districts in Bali have widespread epidemiologic properties. Moreover, CVS that was the reference virus standard from the AAHL share common genetic traits (Figure-3). These data are in line with other reported data showing that rabies viruses within Indonesia share high (98%–99%) genetic homology [14]. The target virus was isolated in this study, as demonstrated by the DFA test and PCR analyses (Figures-1, 2, and 3), although we encountered some problems. At first, we failed to propagate the rabies virus originating from dog brain when it was directly inoculated into N2A cells. However, when the virus from the dog brain samples was first propagated in mice and then inoculated into N2A cells, the virus grew robustly in the cells. Inoculation of naïve mice with the highest viral dilution (10^−4^ cells/mL) of rabies virus-positive tissue culture fluids killed all mice, but the lowest viral concentration (10^−6^ cells/mL) only killed 50% of the infected animals, suggesting that the median lethal dose, 50% of the culture was 10^−6^ cells/mL. These data are crucial when implementing vaccine studies.

## Conclusion

This study provides information regarding the wide epidemiological spread of rabies virus in all districts of Bali. The successful adaptation of the isolated field rabies virus using *in vivo* and *in vivo* studies strongly supports the findings of rabies virus studies in Bali, Indonesia, which may be relevant for developing homologous and feasible vaccine candidates. One limitation of the present study is that the isolated virus was not adapted in other cell lines to obtain a fully attenuated viral antigen and other diagnostic tools, such as monoclonal antibody production and serum neutralization tests.

## Authors’ Contributions

WMT, NSt, MD, IAPA, TKS, and KKA: Designed and conducted the study, analyzed data, and drafted the manuscript. YM, LPA, and NSs: Performed PCR, DFA and tissue culture works, analyzed the data, and drafted and revised the manuscript. All authors have read, reviewed, and approved the final manuscript.
